# Molecular epidemiology and antimicrobial susceptibility of group A Streptococcus isolated from children in Greece during 2023

**DOI:** 10.1007/s15010-025-02639-0

**Published:** 2025-11-03

**Authors:** Elizabeth Barbara Tatsi, Charilaos Dellis, Maria Myrto Dourdouna, Aspasia Rizou, George Paradeisis, Foteini I Koutouzi, Theano Georgakopoulou, Angeliki Stathi, Anastassios Doudoulakakis, George Kalogeras, Levantia Zachariadou, Konstantina Kontopoulou, Athanasios Michos

**Affiliations:** 1 Department of Pediatrics, Infectious Diseases and Chemotherapy Research Laboratory, Medical School, National and Kapodistrian University of Athens, “Aghia Sophia” Children’s Hospital, Athens, 11527 Greece; 2 Department for Vaccine Preventable Diseases and Congenital Diseases, National Public Health Organization (NPHO), Athens, 15123 Greece; 3 Microbiology Department, “Aghia Sophia” Children’s Hospital, Athens, 11527 Greece; 4 Microbiology Laboratory, “P. & A. Kyriakou” Children’s Hospital, Athens, 11527 Greece; 5 Laboratory of Microbiology, “G.Gennimatas” Hospital of Thessaloniki, Thessaloniki, 54635 Greece

**Keywords:** *Streptococcus pyogenes*, *Emm* typing, Children, Antimicrobial resistance profile, Invasive disease

## Abstract

**Purpose:**

This study aimed to describe the molecular epidemiology and antimicrobial susceptibility profiles of invasive (iGAS) and non-invasive (non-iGAS) Group A Streptococcus (GAS) isolates collected from Greek children, including all the Greek fatal pediatric GAS infections, in 2023.

**Methods:**

GAS isolates were prospectively collected from children (0–16 years) with iGAS and non-iGAS infections from January to December 2023. Antimicrobial susceptibility was examined with the disk diffusion method and the MIC of resistant isolates was determined. *Emm* typing was performed in all isolates. Whole genome analysis was performed on *emm1* GAS isolates collected from fatal cases.

**Results:**

GAS isolates from 510 children, with median (IQR) age: 67.8 (46.1–96.0) months, were analyzed in the study. There were 30 (5.9%) iGAS cases, of which nine were fatal. All isolates were penicillin-susceptible, while the resistance rates to tetracycline, erythromycin, and clindamycin were 16.9%, 11.6% and 5.1%, respectively. The M, cMLSB and iMLSB phenotypes were found in 33/510 (6.5%), 22/510 (4.3%) and 4/510 (0.8%) isolates, respectively. Thirty-two *emm* types were detected, with the most prevalent being *emm12* (41.0%), *emm1* (26.9%) and *emm89* (7.5%). Among the different *emm* types, *emm1* was marginally associated with iGAS. The *emm12* type was associated with resistance to clindamycin (*p* = 0.039). GAS isolates from the nine deceased children p-value were identified as *emm1* (7/9), of which 6/7 belonged to M1_UK_ lineage, and *emm12* (2/9).

**Conclusion:**

A predominance of *emm12* and *emm1* was detected in non-iGAS isolates and of *emm1* in iGAS isolates, and specifically M1_UK_ in fatal isolates. A decline in GAS macrolide resistance, compared to previous studies in our area, was detected.

**Supplementary Information:**

The online version contains supplementary material available at 10.1007/s15010-025-02639-0.

## Introduction

*Streptococcus pyogenes* (Group A Streptococcus, GAS), a human-restricted bacterial pathogen, possesses a wide range of virulence determinants involved in the pathogenesis and diverse clinical presentation of the infection ranging from asymptomatic carriage, pharyngitis and scarlet fever to life-threatening invasive disease and immune-mediated complications [1, 2]. This pathogen remains a significant cause of morbidity and mortality, as GAS pharyngitis accounts for 15–30% of cases in children and 5–15% of cases in adults and more than 500,000 deaths annually can be attributed to severe GAS infections worldwide [3–6]. Despite the high global disease burden due to GAS infections, to date a GAS vaccine licenced for commercial use is not available [7, 8]. The development of a GAS vaccine remains challenging mainly due to the pathogen’s extensive genetic diversity and potential of causing autoimmune sequelae [8]. The most advanced multivalent protein-based vaccine, the 30-valent Strep A vaccine (StreptAnova™) targets the GAS surface-expressed protein M, a key virulence factor that assists the pathogen to evade host immune mechanisms, is currently under a clinical trial [8–10].

Antimicrobial treatment is the primary method of controlling and treating GAS infection [11]. Although GAS remains universally susceptibile to penicillin, macrolide resistant strains have been detected in many countries raising concerns, given that macrolides are the first line treatment for patients allergic to β-lactams [12]. The mechanisms of macrolide resistance include antibiotic target site modification by methyltransferases encoded by the *erm* genes (*ermA* and *ermB*) and an active efflux pump encoded by the *mef* gene [12]. GAS isolates harboring the *ermA* gene have an “inducible” macrolides, lincosamides and streptogramins B (MLS_Β_) resistance phenotype (iMLS_Β_), as exposure to a macrolide is required to confer resistance to lincosamides and streptogramins B [13, 14]. The *ermB* gene is primarily associated with a constitutive MLS_Β_ (cMLS_B_) phenotype leading to resistance to all these compounds [12, 13, 15]. The *mefA* gene provides low to moderate resistance to 14- and 15-membered macrolides, whereas GAS isolates carrying this gene remain susceptible to 16-membered MLS _B_ [12, 16].

The onset of the COVID-19 pandemic and the implementation of infection control measures to mitigate SARS-CoV-2, resulted in a reduction in the reported cases of scarlet fever and invasive GAS (iGAS) infections [2, 17, 18]. However, after the lifting of COVID-19-related social distancing measures, a notable rise in the number of cases of GAS pharyngitis, scarlet fever and iGAS infections was observed in the United Kingdom (UK) in late 2022 compared to previous years [2]. Subsequently, a similar rise in GAS infections was rapidly described in several other countries across Europe including France, the Netherlands, Portugal and Denmark and worldwide [2, 5, 9, 18–22]. According to the World Health Organization (WHO), children under the age of 10 years were mostly affected by this upsurge in GAS infection [5]. Furthermore, the number of deaths related to iGAS infections, in some of the countries, also marked an increase [5].

It has been hypothesized that this rise in GAS cases is a result of the lack of immunity against the pathogen, due to the decreased exposure to GAS strains, particularly of children, during the COVID-19 pandemic [6, 20]. Moreover, the increased circulation of respiratory viruses such as seasonal Influenza and Respiratory Syncytial Virus (RSV) after the pandemic might have also contributed to the increase of iGAS cases [6, 20, 21].

GAS bacterial isolates can be classified based on the sequence variation of the 5′ end of the *emm* gene that encodes the M protein [8–10]. To date using *emm* typing, more than 250 distinct *emm* types have been documented [2, 10]. Given that certain *emm* types may be linked to specific GAS virulence profiles and clinical manifestations, *emm* typing is a valuable tool for the epidemiological surveillance of GAS infection [20, 23, 24]. Amid the rise of GAS infections in several countries after the COVID-19 pandemic, genomic surveillance of the GAS strains with *emm* typing, in specific geographic areas, is important to identify the circulation of certain *emm* types and emerging *emm* types, that might have contributed to this upsurge.

The present study aimed to describe the molecular epidemiology and antimicrobial susceptibility patterns of GAS isolates collected from children with GAS infections after the acute phase of the COVID-19 pandemic, including all the reported GAS fatal pediatric cases.

## Materials and methods

### Study participants and bacterial isolates

This is a prospective study that included GAS isolates, collected from children aged ≤ 16 years old who were diagnosed with non-iGAS or iGAS infections and visited the emergency departments or were hospitalized at the pediatric hospitals in Athens and Thessaloniki, Greece, from January to December 2023. Cultures were sent to the reference laboratory “Infectious Diseases and Chemotherapy” of the First Department of Pediatrics of “Aghia Sophia” Children’s Hospital, Athens, Greece for further testing.

Demographic (sex, age, ethnicity) and epidemiological (type of infection, isolation site, sample collection date, disease outcome) data from the children were also collected. Children were classified according to their age in the following age-groups: infants (< 1 year), toddlers (1 - < 3 years), preschoolers (3 - < 6 years), school-age children (6 - < 12 years) and adolescents (12 - ≤ 16 years).

A case of iGAS infection was defined as an illness associated with isolation of GAS by culture or detection of GAS by molecular testing from a normally sterile site (e.g. blood, cerebrospinal fluid, joint fluid, peritoneal fluid, bone, internal organs) [25]. In addition, cases of septic shock, Streptococcal Toxic Shock Syndrome (STSS), or necrotizing fasciitis, for which no other bacterial etiology was identified and in which GAS was isolated or detected from a nonsterile site (e.g. throat, sputum, wound, superficial skin abscess, subcutaneous tissue) were also considered to be iGAS infections [25].

GAS isolates were confirmed by colony morphology, typical β-haemolysis on 5% sheep blood agar (Becton Dickinson, Franklin Lakes, NJ, USA) and inhibition screening test using 0.04 U bacitracin disks (OXOID Ltd, Basingstoke, UK). The GAS isolates were stored at -80 ^ο^C.

The study was carried out in accordance with the Declaration of Helsinki and the study protocol was approved by the scientific and bioethics committee of “Aghia Sophia” Children’s Hospital (No. 21027).

### Antimicrobial susceptibility testing

Antimicrobial resistance profile for seven antibiotics was assessed in all isolates using the disk diffusion method (OXOID Ltd, Basingstoke, UK) according to the European Committee on Antimicrobial Susceptibility Testing (EUCAST) guidelines (https://www.eucast.org/). In GAS isolates that were found to be resistant with the disk diffusion method, the Minimum Inhibitory Concentration (MIC) was also determined, using the MIC Test Strip (MTS; Liofilchem, Teramo, Italy). The tested antibiotics were erythromycin [ERY; 15 mcg and 0.016–256 µg/mL], clindamycin [CLI; 2 mcg and 0.016–256 µg/mL], tetracycline [TET; 30 mcg and 0.016–256 µg/mL], benzylpenicillin [BEN; 10 IU and 0.002–32 µg/mL], moxifloxacin [MOX; 5 mcg and 0.002–32 µg/mL], rifampicin [RIF; 5 mcg and 0.002–32 µg/mL], and vancomycin [VAN; 30 mcg and 0.016–256 µg/mL].

To examine the macrolide resistance phenotypes, the double-disk diffusion test (D-zone or D-test) was performed in erythromycin-resistant GAS isolates, using 15 mcg erythromycin disks and 2 mcg clindamycin disks placed 12 mm apart on Mueller-Hinton 5% sheep blood agar plates [26]. Isolates with flattening of the clindamycin inhibition zone in proximity to the erythromycin disk (positive D-test) had the inducible Macrolide-Lincosamide-Streptogramin B resistance phenotype (iMLS_B_) [26–28]. Clindamycin-susceptible isolates without flattening of the inhibition zone had the M resistance phenotype (resistance to macrolides alone) [27, 28]. The isolates that were resistant to both erythromycin and clindamycin had the constitutive Macrolide-Lincosamide-Streptogramin B resistance phenotype (cMLS_B_) [27, 28].

### DNA isolation and *emm* typing

Bacterial genome isolation from culture was performed with the Maxwell RSC Cultured Cells DNA kit (Promega, Madison, WI, USA) according to manufacturer’s instructions. The guidelines of the Centers for Disease Control and Prevention (CDC) were followed for the amplification of hypervariable sequence of *emm* gene with polymerase chain reaction (PCR) method [29]. Sanger sequencing was performed using the BigDye Terminator v3.1 cycle sequencer kit on an Applied Biosystems 3500 genetic analyzer (Applied Biosystems, Waltham, MA, USA). For the determination of *emm* type and *emm* subtypes, the CDC blast 2.0 (https://www2.cdc.gov/vaccines/biotech/strepblast.asp) were used.

### Whole genome sequencing

FASTQ data was assessed for quality and filtered using filtlong tool. Trimming was performed with the command line tool porechop (https://github.com/rrwick/Porechop), that finds and removes the adapters, and *de novo* assembly for single-molecule sequencing reads was carried out with the command line tool flye (https://github.com/mikolmogorov/Flye). The assembled FASTA files were uploaded to Pathogenwatch (https://pathogen.watch/) for organism identification. *Emm* types were determined with the emmtyper command line tool (https://github.com/MDU-PHL/emmtyper). The Virulence Factor Database (VFDB) was employed for the annotation of known virulence-associated genes (https://www.mgc.ac.cn/VFs/). The command line tools ResFinder, Comprehensive Antibiotic Resistance Database (CARD) and ARG-ANNOT through ABricate (https://github.com/tseemann/abricate) were used for antimicrobial resistance (AMR) gene identification.

To identify whether the strains belonged to the M1_UK_ or M1_DK_ lineage, the *emm1* GAS genomes from this study were compared with the reference genome MGAS5005 (GenBank accession no. NC_007297.2) using the snippy command line tool (https://github.com/tseemann/snippy), that performs variant calling and core genome alignment. According to Lynskey et al. (2019), 27 specific single nucleotide polymorphisms (SNPs) distinguish M1_UK_ from M1_global_ lineages and according to Johannesen et al. (2023) 15 specific SNPs distinguish M1_DK_ from M1_global_ lineages [30, 31].

FASTQ genome data were submitted to the NCBI database Sequence Read Archive (SRA) https://www.ncbi.nlm.nih.gov/sra.

### Statistical analysis

Absolute and relative frequencies (%) were used to describe qualitative variables, and median and interquartile range (IQR) were used to describe quantitative data. Comparisons of the qualitative parameters were performed using Fisher’s exact test or Pearson’s chi-squared test and Monte Carlo method. Comparisons of the quantitative parameters were performed using the Mann-Whitney U test. The statistical significance level was set at 0.05. Statistical analysis was performed using SPSS version 26.0 (IBM Corp., Released 2019. IBM SPSS Statistics for Windows, Version 26.0. Armonk, NY: IBM Corp). Additionally, the diversity of the emm-types was examined through Simpson’s index of diversity with the use of an online tool (https://virtue.gmbl.se/english-content/biodiversity-calculator). Corrections for multiple comparisons were performed with the Bonferroni test also using an online tool (https://www.statology.org/bonferroni-correction-calculator/). Figures were created using GraphPad Prism Software Version 10.1.1 (270) (GraphPad Software Inc., San Diego, CA, USA).

## Results

### Demographic and clinical characteristics of the study population

A total of 510 children with GAS infection were included in the study. iGAS cases represented 5.9% (30/510) and 9/510 (1.8%) were fatal. The epidemiological characteristics of the study population are presented in Table 1. The median (IQR) age of children was 67.8 (46.1–96.0) months, 288/510 (56.5%) were males and 378/510 (74.1%) were of Greek origin. Most of them were school-age children (198/496, 39.9%) and pre-school-age children (179/496, 36.1%). The highest number of iGAS and non-iGAS cases occurred in school-age children [12/30, 40.0% and 186/466, 39.9%, respectively]. Children with iGAS infection were older [median (IQR) 68.2 (47.0–96.0) months] than children with non-iGAS infection [median (IQR) 55.0 (30.6–82.5) months], (*p* = 0.054) (Table 1). No statistically significant association was detected between invasive disease and age groups (*p* = 0.06). The most common clinical presentations in children with iGAS infection were bacteremia (23/30, 76.7%) and STSS (9/30, 30.0%). The majority of the children with non-iGAS infection presented with pharyngitis (294/480, 61.3%) and otitis (83/480, 17.3%) (Table 1).

**Table 1:** Demographic and clinical characteristics of children with Group A Streptococcus (GAS) invasive (iGAS) and non-invasive (non-iGAS) infection during 2023 in Greece (*n* = 510)

	Study Population No. of participants (%)	iGAS infection No. of participants (%)	non-iGAS infection No. of participants (%)	*p*-value
**Gender (male)**	288/510 (56.5)	17/30 (56.7)	271/480 (56.5)	0.999
**Age Group**				
Infants (< 1y)	18/496 (3.6)	1/30 (3.3)	17/466 (3.6)	0.999
Toddlers (1-<3y)	68/496 (13.7)	9/30 (30.0)	59/466 (12.7)	0.013
Pre-schoolers (3-<6y)	179/496 (36.1)	8/30 (26.7)	171/466 (36.7)	0.329
School-age children (6-<12y)	198/496 (39.9)	12/30 (40.0)	186/466 (39.9)	0.999
Adolescents (12-≤16y)	32/496 (6.5)	0/30 (0.0)	32/466 (6.9)	0.246
**Origin (Greek)**	378/510 (74.1)	24/30 (80.0)	354/480 (73.8)	0.526
Type of infection*				
Pharyngitis	294/510 (57.6)	0/30 (0.0)	294/480 (61.3)	**< 0.001**
Otitis	83/510 (16.3)	0/30 (0.0)	83/480 (17.3)	**0.008**
Peritonsillar abscess	40/510 (7.8)	0/30 (0.0)	40/480 (8.3)	0.156
Bacteremia	23/510 (4.5)	23/30 (76.7)	0/480 (0.0)	**< 0.001**
Pneumonia	10/510 (2.0)	10/30 (33.3)	0/480 (0.0)	**< 0.001**
Mastoiditis	3/510 (0.6)	3/30 (10.0)	0/480 (0.0)	**< 0.001**
Osteomyelitis	2/510 (0.4)	2/30 (6.7)	0/480 (0.0)	**0.003**
Arthritis	3/510 (0.6)	3/30 (10.0)	0/480 (0.0)	**< 0.001**
Skin and Soft Tisue Infections	57/510 (11.2)	3/30 (10.0)	54/480 (11.3)	0.999
STSS	9/510 (1.8)	9/30 (30.0)	0/480 (0.0)	**< 0.001**
Vaginitis	5/510 (1.0)	0/30 (0.0)	5/480 (1.0)	0.999
Conjunctivitis	2/510 (0.4)	0/30 (0.0)	2/480 (0.4)	0.999
UTI	2/510 (0.4)	0/30 (0.0)	2/480 (0.4)	0.999
Meningitis	2/510 (0.4)	2/30 (6.7)	0/480 (0.0)	0.003
**Fatal outcome**	9/510 (1.8)	9/30 (30.0)	0/480 (0.0)	**< 0.001**

Notes: Values are referred as relative frequencies (%) or * Median and IQR. *p*-value obtained after conducting Fisher’s exact test. Statistically significant differences (*p*-value < 0.05) are marked in bold

Abbreviations: n/a = non applicable, UTI = Urinary Tract Infection; * children with iGAS infection may have more than one clinical syndrome

### GAS antimicrobial susceptibility testing

GAS antimicrobial resistance was detected to tetracycline (86/510, 16.9%), erythromycin (59/510, 11.6%), clindamycin (26/510, 5.1%), rifampicin (30/510, 5.9%), and moxifloxacin (17/510, 3.3%) (Table 2). In GAS isolates that were found to be resistant with the disk diffusion method, the MIC was also determined, based on the EUCAST breakpoints. Specifically, in resistant isolates the MIC range was for tetracycline 1.5–48 µg/mL, for erythromycin 0.38–256 µg/mL, for clindamycin 1.5–256 µg/mL, for rifampicin 0.094-0.38 µg/mL and for moxifloxacin 1–2 µg/mL. The M phenotype was found in 33/510 (6.5%) of GAS isolates, three of which were iGAS. Moreover, 22/510 (4.3%) isolates had the constitutive resistance cMLSB phenotype, of which one isolate was iGAS. Inducible resistance to clindamycin (iMLSB phenotype) was found in 4/510 (0.8%) of these isolates, all of which were non-iGAS.

**Table 2:** Antimicrobial resistance to seven antimicrobial agents of group A Streptococcus (GAS) isolates collected from children with GAS infections during 2023 in Greece

Antimicrobial agent	GAS isolates	iGAS isolates	non-iGAS isolates	*p*-value
Tetracycline	86/510 (16.9)	4/30 (13.3)	82/480 (17.1)	0.802
Erythromycin	59/510 (11.6)	4/30 (13.3)	55/480 (11.5)	0.767
M-phenotype	33/59 (55.9)	3/4 (75.0)	30/55 (54.5)	0.623
cMLS_**B**_ phenotype	22/59 (37.3)	1/4 (25.0)	2155 (38.2)	0.999
iMLS_B_ phenotype	4/59 (6.8)	0/4 (0)	4/55 (7.3)	0.999
Clindamycin	26/510 (5.1)	1/30 (3.3)	25/480 (5.2)	0.999
Rifampicin	30/510 (5.9)	1/30 (3.3)	29/480 (6.0)	0.999
Moxifloxacin	17/510 (3.3)	1/30 (3.3)	16/480 (3.3)	0.999
Vancomycin	0/510 (0.0)	0/30 (0.0)	0/480 (0.0)	n/a
Penicillin	0/510 (0.0)	0/30 (0.0)	0/480 (0.0)	n/a

Notes: Values are referred to as absolute frequencies (relative frequencies, %). *p*-value obtained after conducting Fisher’s exact test.

Abbreviations: M-phenotype = resistance only to Macrolides, cMLS_B_ = constitutive Macrolide-Lincosamide-Streptogramin B resistant resistance phenotype (cMLS_B_) iMLS_B_ = inducible Macrolide-lincosamide-streptogramin B resistance phenotype, n/a = non applicable

### Distribution of GAS *emm* types

Thirty-two distinct *emm* types and 64 different *emm* subtypes were detected (Tables 3 and 4). The most common *emm* types were *emm12* (209/510, 41.0%), *emm1* (137/510, 26.9%), *emm89* (38/510, 7.5%) and *emm75* (23/510, 4.5%) and the most common subtypes were *emm12.0* (144/510, 28.2%), *emm1.0* (119/510, 23.3%) and *emm12.101* (37/510, 7.3%).

**Table 3:** Distribution of the *emm* types of Group A Streptococcus (GAS) isolates collected from children with invasive (iGAS) (*n* = 30) and non-invasive (non-iGAS) (*n* = 480) infection during 2023 in Greece

*Emm* type	No. of GAS isolates (*N* = 510)	iGAS isolates (*N* = 30)	non-iGAS isolates (*N* = 480)	*p*-value
*emm1*	137 (26.9)	16 (53.3)	121 (25.2)	**0.002**
*emm2*	6 (1.2)	0 (0.0)	6 (1.3)	0.999
*emm3*	4 (0.8)	0 (0.0)	4 (0.8)	0.999
*emm4*	6 (1.2)	0 (0.0)	6 (1.3)	0.999
*emm8*	2 (0.4)	0 (0.0)	2 (0.4)	0.999
*emm11*	1 (0.2)	0 (0.0)	1 (0.2)	0.999
*emm12*	209 (41.0)	11 (36.7)	198 (41.3)	0.704
*emm22*	2 (0.4)	0 (0.0)	2 (0.4)	0.999
*emm28*	20 (3.9)	0 (0.0)	20 (4.2)	0.622
*emm33*	2 (0.4)	0 (0.0)	2 (0.4)	0.999
*emm43*	5 (1.0)	0 (0.0)	5 (1.0)	0.999
*emm44*	1 (0.2)	0 (0.0)	1 (0.2)	0.999
*emm49*	3 (0.6)	0 (0.0)	3 (0.6)	0.999
*emm60*	3 (0.6)	0 (0.0)	3 (0.6)	0.999
*emm63*	1 (0.2)	0 (0.0)	1 (0.2)	0.999
*emm68*	1 (0.2)	1 (3.3)	0 (0.0)	0.059
*emm73*	1 (0.2)	0 (0)	1 (0.2)	0.999
*emm75*	23 (4.5)	1 (3.3)	22 (4.6)	0.999
*emm76*	4 (0.8)	0 (0.0)	4 (0.8)	0.999
*emm77*	4 (0.8)	0 (0.0)	4 (0.8)	0.999
*emm81*	1 (0.2)	0 (0.0)	1 (0.2)	0.999
*emm82*	1 (0.2)	0 (0.0)	1 (0.2)	0.999
*emm83*	1 (0.2)	0 (0.0)	1 (0.2)	0.999
*emm87*	4 (0.8)	0 (0.0)	4 (0.8)	0.999
*emm89*	38 (7.5)	1 (3.3)	37 (7.7)	0.716
*emm104*	4 (0.8)	0 (0.0)	4 (0.8)	0.999
*emm118*	2 (0.4)	0 (0.0)	2 (0.4)	0.999
*emm128*	20 (3.9)	0 (0.0)	20 (4.2)	0.622
*emm168*	1 (0.2)	0 (0.0)	1 (0.2)	0.999
*emm223*	1 (0.2)	0 (0.0)	1 (0.2)	0.999
*emm227*	1 (0.2)	0 (0.0)	1 (0.2)	0.999
*emm228*	1 (0.2)	0 (0.0)	1 (0.2)	0.999

Notes: Values are referred to as absolute frequencies (relative frequencies, %). *p*-value obtained after conducting Fisher’s exact test. Statistically significant differences (*p*-value < 0.05) are marked in bold

**Table 4:** *Emm* type and subtype distribution detected in 510 Group A Streptococcus (GAS) isolates collected from children with GAS infections from January to December 2023 in Greece

*Emm* type	No. (%) of isolates	*emm*-subtype	No. (%) of isolates	No. (%) of iGAS	No. (%) of non-iGAS
*emm1*	137 (26.9)	*emm1.0*	119 (23.3)	14 (46.7)	105 (21.9)
		*emm1.169*	4 (0.8)	0 (0.0)	4 (0.8)
		*emm1.44*	3 (0.6)	0 (0.0)	3 (0.6)
		*emm1.29*	2 (0.4)	0 (0.0)	2 (0.4)
		*emm1.38*	2 (0.4)	0 (0.0)	2 (0.4)
		*emm1.137*	1 (0.2)	1 (3.3)	0 (0.0)
		*emm1.143*	1 (0.2)	0 (0.0)	1 (0.2)
		*emm1.46*	1 (0.2)	0 (0.0)	1 (0.2)
		*emm1.55*	1 (0.2)	0 (0.0)	1 (0.2)
		*emm1.61*	1 (0.2)	1 (3.3)	0 (0.0)
		*emm1.66*	1 (0.2)	0 (0.0)	1 (0.2)
		*emm1.76*	1 (0.2)	0 (0.0)	1 (0.2)
*emm2*	6 (1.2)	*emm2.0*	6 (1.2)	0 (0.0)	6 (1.3)
*emm3*	4 (0.8)	*emm3.93*	4 (0.8)	0 (0.0)	4 (0.8)
*emm4*	6 (1.2)	*emm4.0*	4 (0.8)	0 (0.0)	4 (0.8)
		*emm4.19*	2 (0.4)	0 (0.0)	2 (0.4)
*emm8*	2 (0.4)	*emm8.0*	1 (0.2)	0 (0.0)	1 (0.2)
		*emm8.5*	1 (0.2)	0 (0.0)	1 (0.2)
*emm11*	1 (0.2)	*emm11.0*	1 (0.2)	0 (0.0)	1 (0.2)
*emm12*	209 (41.0)	*emm12.0*	144 (28.2)	4 (13.3)	140 (29.2)
		*emm12.1*	1 (0.2)	0 (0.0)	1 (0.2)
		*emm12.101*	37 (7.3)	5 (16.7)	32 (6.7)
		*emm12.133*	5 (1.0)	0 (0.0)	5 (1.0)
		*emm12.22*	1 (0.2)	0 (0.0)	1 (0.2)
		*emm12.37*	4 (0.8)	0 (0.0)	4 (0.8)
		*emm12.40*	6 (1.2)	2 (6.7)	4 (0.8)
		*emm12.41*	2 (0.4)	0 (0.0)	2 (0.4)
		*emm12.5*	1 (0.2)	0 (0.0)	1 (0.2)
		*emm12.62*	1 (0.2)	0 (0.0)	1 (0.2)
		*emm12.76*	7 (1.4)	0 (0.0)	7 (1.5)
*emm22*	2 (0.4)	*emm22.0*	2 (0.4)	0 (0.0)	2 (0.4)
*emm28*	20 (3.9)	*emm28.0*	16 (3.1)	0 (0.0)	16 (3.3)
		*emm28.3*	1 (0.2)	0 (0.0)	1 (0.2)
		*emm28.32*	3 (0.6)	0 (0.0)	3 (0.6)
*emm33*	2 (0.4)	*emm33.0*	2 (0.4)	0 (0.0)	2 (0.4)
*emm43*	5 (1.0)	*emm43.0*	1 (0.2)	0 (0.0)	1 (0.2)
		*emm43.4*	4 (0.8)	0 (0.0)	4 (0.8)
*emm44*	1 (0.2)	*emm44.0*	1 (0.2)	0 (0.0)	1 (0.2)
*emm49*	3 (0.6)	*emm49.1*	1 (0.2)	0 (0.0)	1 (0.2)
		*emm49.3*	2 (0.4)	0 (0.0)	2 (0.4)
*emm60*	3 (0.6)	*emm60.11*	3 (0.6)	0 (0.0)	3 (0.6)
*emm63*	1 (0.2)	*emm63.3*	1 (0.2)	0 (0.0)	1 (0.2)
*emm68*	1 (0.2)	*emm68.3*	1 (0.2)	1 (3.3)	0 (0.0)
*emm73*	1 (0.2)	*emm73.0*	1 (0.2)	0 (0.0)	1 (0.2)
*emm75*	23 (4.5)	*emm75.0*	22 (4.3)	1 (3.3)	21(4.4)
		*emm75.1*	1 (0.2)	0 (0.0)	1 (0.2)
*emm76*	4 (0.8)	*emm76.0*	4 (0.8)	0 (0.0)	4 (0.8)
*emm77*	4 (0.8)	*emm77.0*	4 (0.8)	0 (0.0)	4 (0.8)
*emm81*	1 (0.2)	*emm81.0*	1 (0.2)	0 (0.0)	1 (0.2)
*emm82*	1 (0.2)	*emm82.1*	1 (0.2)	0 (0.0)	1 (0.2)
*emm83*	1 (0.2)	*emm83.1*	1 (0.2)	0 (0.0)	1 (0.2)
*emm87*	4 (0.8)	*emm87.0*	4 (0.8)	0 (0.0)	4 (0.8)
*emm89*	38 (7.5)	*emm89.0*	33 (6.5)	1 (3.3)	32 (6.7)
		*emm89.2*	1 (0.2)	0 (0.0)	1 (0.2)
		*emm89.22*	2 (0.4)	0 (0.0)	2 (0.4)
		*emm89.28*	1 (0.2)	0 (0.0)	1 (0.2)
		*emm89.34*	1 (0.2)	0 (0.0)	1 (0.2)
*emm104*	4 (0.8)	*emm104.0*	4 (0.8)	0 (0.0)	4 (0.8)
*emm118*	2 (0.4)	*emm118.0*	2 (0.4)	0 (0.0)	2 (0.4)
*emm128*	20 (3.9)	*emm128.0*	20 (3.9)	0 (0.0)	20 (4.2)
*emm168*	1 (0.2)	*emm168.1*	1 (0.2)	0 (0.0)	1 (0.2)
*emm223*	1 (0.2)	*emm223.0*	1 (0.2)	0 (0.0)	1 (0.2)
*emm227*	1 (0.2)	*emm227.1*	1 (0.2)	0 (0.0)	1 (0.2)
*emm228*	1 (0.2)	*emm228.0*	1 (0.2)	0 (0.0)	1 (0.2)

Notes: Values are referred to as absolute frequencies (relative frequencies, %)

**Fig. 1:**
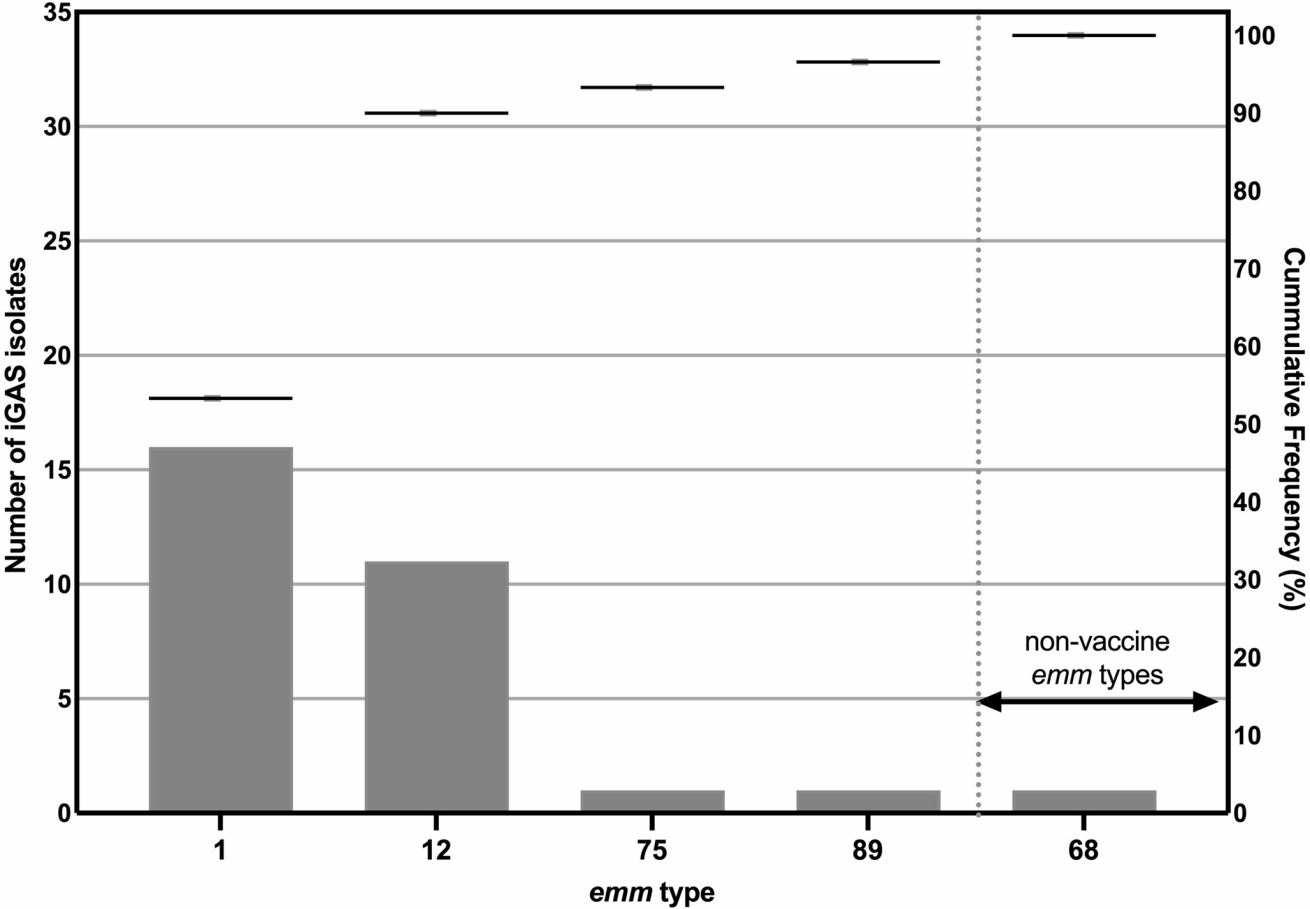
Distribution and cumulative frequencies of *emm* types detected in 30 invasive Group A Streptococcus (iGAS) isolated from children during 2023. The chart displays the *emm* types included in the proposed 30-valent GAS vaccine on the left, and those not included in the vaccine on the right

In the iGAS group, 5 different *emm* types were identified and the most common *emm* types were *emm1 (*16/30, 53.3%) and *emm12* (11/30, 36.7%), (Fig. 1). The type *emm68* was exclusively found in the iGAS group. In the non-iGAS group, 31 distinct *emm* types were detected, and the most common *emm* types were *emm12 (*198/480, 41.3%) followed by *emm1* (121/480, 25.2%), *emm89* (37/480, 7.7%), *emm28* (20/480, 4.2%) and *emm128* (20/480, 4.2%). Among isolates from pharyngitis cases, the most common type was *emm12* (136/294, 46.3%), followed by *emm1* (67/294, 22.8%). The *emm* types 2, 3, 4, 8, 11, 22, 28, 33, 43, 44, 49, 60, 63, 73, 76, 77, 81, 82, 83, 87, 104, 118, 128, 168, 223, 227, and 228 were found only in the non-iGAS group.

**Fig. 2:**
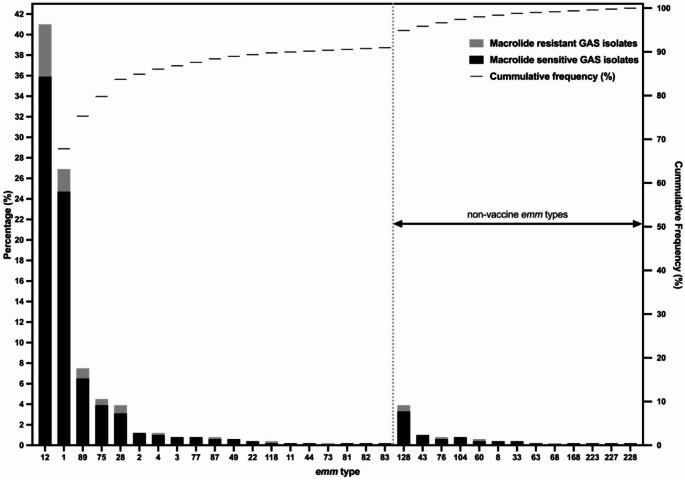
Distribution and cumulative frequencies of the *emm* types detected among 510 Group A Streptococcus (GAS) isolates collected from children during 2023. Gray colored bars represent the percentage of macrolide resistant isolates, while black colored bars represent the percentage of macrolide sensitive isolates. The *emm* types included in the proposed 30-valent GAS vaccine are shown on the left side of the chart and the non-vaccine *emm* types on the right side

To compare the diversity between iGAS and non-iGAS isolates, Simpson’s Index of Diversity was calculated, yielding values of 0.74 for the total sample, 0.57 for iGAS isolates, and 0.75 for non-iGAS isolates. Statistical differences between the iGAS and the non-iGAS isolates were found in *emm1* (53.3% of *emm1* isolates in iGAS cases vs. 25.2% of *emm1* isolates in non-iGAS cases, *p =* 0.002) (Table 3). After applying the Bonferroni correction for multiple comparisons (adjusted significance level set at *p* < 0.0016), the above result was found marginally statistically significant.

The associations of the five most common *emm* subtypes (*emm1.0*, *emm12.0*, *emm12.101*, *emm28.0* and *emm89.0*) and invasive disease were examined. Statistical differences between the iGAS and the non-iGAS isolates were found in *emm12.0* isolates (36.4% of *emm12.0* isolates in iGAS cases caused by *emm12* vs. 70.7% of *emm12.0* isolates in non-iGAS cases caused by emm12, *p =* 0.038) and in *emm12.101* isolates (45.5% of *emm12.101* isolates in iGAS cases caused by *emm12* vs. 16.2% of *emm12.101* isolates in non-iGAS cases *caused by emm12*, *p =* 0.027).

Twenty-nine *emm* types belonged to nine discrete clusters. The most frequent clusters were A-C4 (209/510, 41.0%), A-C3 (137/510, 26.9%), E4 (73/510, 14.3%) and E6 (26/510, 5.1%) accounting for 87.3% of all the GAS isolates (Table 5). Three clusters (A-C4, A-C3, A-C5) included only one *emm* type. Cluster A-C3 was associated with iGAS infections (*p* = 0.002). Based on the Bonferroni correction for multiple comparisons (adjusted significance level set at *p* < 0.0056), the above result remains statistically significant.

**Table 5:** Distribution of the *emm* types of Group A Streptococcus isolates collected from 510 children with *Streptococcus pyogenes* infections during 2023 according to *emm* clusters

*emm* cluster	*emm* types	No. (%) of isolates	iGAS infection No. (%)	non-iGAS infection No. (%)	*p*-value
A-C4	12	209 (41.0)	11 (36.7)	198 (41.3)	0.704
A-C3	1	137 (26.9)	16 (53.3)	121 (25.2)	**0.002**
E4	2, 8, 22, 28, 73, 77, 89	73 (14.3)	1 (3.3)	72 (15.0)	0.103
E6	11, 63, 75, 81	26 (5.1)	1 (3.3)	25 (5.2)	0.999
E3	44, 49, 82, 87, 118	11 (2.2)	0 (0.0)	11 (2.3)	0.999
E2	68, 76, 104, 168	10 (2.0)	1 (3.3)	9 (1.9)	0.458
D4	33, 43, 83, 223	9 (1.8)	0 (0.0)	9 (1.9)	0.999
E1	4, 60	9 (1.8)	0 (0.0)	9 (1.9)	0.999
A-C5	3	4 (0.8)	0 (0.0)	4 (0.8)	0.999

Notes: Values are referred to as absolute frequencies (relative frequencies, %). *p*-value obtained after conducting Fisher’s exact test. Statistically significant differences (*p*-value < 0.05) are marked in bold

The *emm* types identified in this study represent 91.0% (464/510) of all GAS isolates [including 53/464 (12.2%) macrolide resistant isolates] and 96.7% (29/30) of iGAS that are included in a proposed 30-valent vaccine (Figs. 1 and 2) [32]. Among the types that were not included in the vaccine, *emm128* (20/510, 3.9%) and *emm43* (5/510, 1.0%) were the most prevalent.

**Fig. 3:**
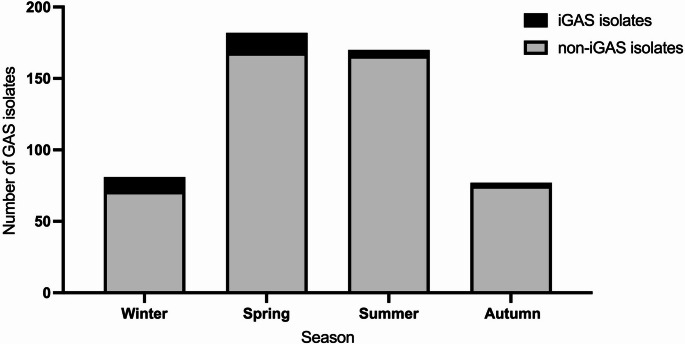
Number of Group A Streptococcus (GAS) invasive (iGAS) and non-invasive (non-iGAS) isolates per season collected from children with streptococcal infections in Greece during 2023

### Associations between the *emm* type and the age groups, seasonality and antimicrobial resistance profile

A statistically significant association between the age groups of the children and the *emm* types of the GAS isolates was observed (*p* = 0.015) (Supplementary Table 1). In all age groups, *emm12* was the predominant GAS isolate followed by *emm1*. Specifically, the most frequent types were *emm12* (21.1%) *emm1* (15.8%) and *emm89* (15.8%) in infants. For toddlers, *emm12* (44.1%) and *emm1* (33.8%) were the most prevalent types. Among pre-school-age children, *emm12* accounted for 41.9% of cases, followed by *emm1* at 25.7%. In school-age children, *emm12* (40.9%) and *emm1* (26.3%) remained the dominant types. Similarly, in adolescents, *emm12* (34.4%) and *emm1* (28.1%) were the most frequent *emm* types identified. Seasonality of GAS infections was also significantly associated with *emm* types (*p* < 0.001) (Fig. 3; Supplementary Table 2). During the winter, *emm1* was the most common type, accounting for 42.0% of cases. In spring and summer, *emm12* was the predominant type, comprising 51.6% and 49.4% of cases, respectively. In autumn, *emm1* (28.6%) was the most prevalent, followed by *emm12* (27.3%) (Supplementary Table 2). The associations between the four most common *emm* types identified in this study and resistance to seven antimicrobial agents, were examined. Isolates of type *emm1* were significantly associated with lower resistance to clindamycin (0.7% of the *emm1* isolates were resistant vs. 6.7% of other *emm* types, *p* = 0.005). In contrast, *emm12* isolates were associated with lower resistance to tetracycline (10.5% of *emm12* vs. 21.3% of other *emm* types, *p* = 0.002) but higher resistance to clindamycin (7.7% of *emm12* vs. 3.3% of other *emm* types, *p* = 0.039). Isolates belonging to *emm89* were linked to higher resistance to moxifloxacin (10.5% vs. 2.8% of other *emm* types, *p* = 0.031). No significant associations with antibiotic resistance were found for *emm75*. Among children with iGAS infection (*n* = 30), the associations between resistance to seven antimicrobial agents and the two most common *emm* types (*emm1*, e*mm12*) identified in iGAS cases were also examined and no statistically significant differences were detected (data not shown).

### Characterization of fatal GAS isolates

The median (IQR) age of the deceased children (*n* = 9) was 31.8 (20.8–77.1) months and most of them were males (6/9, 66.7%). The clinical syndromes and virulence factors genes of the *emm1* strains isolated from fatal infections are presented in Supplementary Table 3. Antimicrobial resistance genes were not detected in any one of these isolates. Among the iGAS isolates collected from the deceased children, only the *emm1* (7/9, 77.8%) and *emm12* (2/9, 22.2%) types were detected. The dominant subtype of fatal GAS isolates *was emm1.0* (6/9, 66.7%), followed by *emm1.61* (1/9, 11.1%), *emm12.0* (1/9, 11.1%) and *emm12.40* (1/9, 11.1%). To further investigate the origin of *emm1* GAS fatal isolates (*n* = 7), WGS were performed. Among *emm1* isolates (*n* = 7), 6/7 isolates (*emm1.0*) belonged to the M1_UK_ lineage and 1/7 isolate (*emm1.61*) belonged to the M1_global_ lineage. None of these isolates belonged to the M1_DK_ lineage.

## Discussion

The current study investigated the molecular epidemiology and antimicrobial susceptibility patterns of GAS isolated from children with non-iGAS and iGAS infections in Greece during 2023, including all the fatal GAS infections. Findings reveal important trends regarding the *emm* type distribution and antimicrobial resistance patterns. While various *emm* types were detected, *emm1* and *emm12* were the most prevalent, including in iGAS isolates and iGAS isolates from fatal cases. The age group mostly affected by GAS infections was school-age children.

In this study, 32 different *emm* types were detected, highlighting the high genetic diversity of GAS. In Greece, the most recent studies analyzing *emm* typing in GAS isolates from the pediatric population were conducted between 2007 and 2013 and 2011–2017 [33, 34]. The first study Koutouzi FI et al. examined over 1,200 isolates and identified 35 distinct *emm* types [33] while the second by Grivea I et al. analyzed 517 isolates and identified 20 *emm* types [34]. In our study, *emm12* was the most prevalent type, followed by *emm1*,* emm89* and *emm75*. In Greece, *emm1*,* emm12*,* emm77* and *emm4* were the four most common *emm* types identified in a 2007–2013 study [33]. Additionally, a higher yearly diversity of *emm* types was found compared Koutouzi’s results to our study [33]. Furthermore, data from 2011 to 2017 revealed that the most prevalent *emm* types included *emm1*,* emm89*,* emm4* and *emm12*. While these variations reflect the temporal diversity of *emm* types, *emm1* and *emm12* have consistently remained among the most common *emm* types circulating in our country, over the past few years.

During the recent surge of GAS infections in late 2022 in Europe, genomic data of GAS isolates collected from patients with both invasive and non-invasive infections in the UK revealed that *emm12* and *emm1* types predominated during the outbreak [20]. In a study from Germany including iGAS and non-iGAS isolates, 33 distinct *emm* types were identified, with *emm1*,* emm89*, and *emm12* being the most prevalent [35]. Similarly to our findings, a study from Italy that was conducted between September 2022 and March 2023 and also included both types of isolates that belonged to 13 distinct *emm* types, found that the most prevalent *emm* types were *emm1* and *emm12* [36]. Altogether, the above findings support, that although multiple *emm* types may have been circulating during this outbreak, *emm12* and *emm1* predominated. As observed in many other countries, in the present study, *emm1* was the most frequent *emm* type identified in iGAS cases [30, 35, 36]. Of note, GAS isolates expressing the M1 protein are recognized as a leading cause of invasive infections globally and infections caused by these GAS isolates, have been associated with the need of intensive care and high case fatality [35, 37]. Regarding specifically non-iGAS infections, the most common *emm* types were *emm12* and *emm1*. In the current study, *emm12* (46.3%) was the most common type in isolates from cases presenting with pharyngitis, followed by *emm1* (22.8%). A UK study conducted during 2022–2023 that included non-invasive throat and skin GAS isolates, found that the most common types were *emm1* (28.7 %) and *emm12* (24.9 %) in throat isolates and *emm1* (22 %), *emm12* (10 %), *emm76* (18 %) in skin isolates [38].

During a previous period of large annual upsurges in scarlet fever and iGAS cases in the UK, between 2014 and 2018, an emerging lineage of *emm1* GAS isolates, designated as M1_UK_, became dominant [2, 31]. A characteristic of this lineage is the increased expression of streptococcal pyrogenic exotoxin A (SpeA) [2]. Subsequently, the M1_UK_ strain spread worldwide and was detected in several other countries including Denmark, the Netherlands, Italy, the United States and Canada and Australia [1, 30, 39–42]. In Greece, a recent report from 2024 described an iGAS case of an adult patient with sepsis with disseminated intravascular coagulation, pneumonia with pleural empyema and streptococcal toxic shock syndrome caused by a M1_UK_ GAS strain [43].

In the present study, all *emm1* GAS isolates collected from fatal pediatric cases underwent whole genome sequencing revealing that the M1_UK_ was the dominant lineage. In line with our findings, during the recent post-COVID-19 GAS outbreak in the UK, in late 2022, a study reported that most of the iGAS infections were caused by *emm1* isolates, the majority of which belonged to the M1_UK_ lineage [20]. Notably, our study is the first to detect the circulation of the M1_UK_ GAS strain among Greek children during 2023 and report on fatal pediatric GAS infections caused by this strain.

While efforts to develop a safe and effective vaccine to reduce the GAS disease burden have been ongoing for years, a vaccine against GAS has not yet been approved for use in clinical practice [8, 32]. In 2011, a multivalent M protein-based vaccine (Strep A vaccine, StreptAnova™) containing N-terminal peptides from 30 M-proteins of frequent pharyngitis, invasive and/or rheumatogenic GAS serotypes was developed and was later found to be well tolerated and immunogenic among participants in a Phase 1 clinical trial [8, 32, 44]. In a previous Greek study, the *emm* types included in the proposed 30-valent vaccine accounted for 98.8% of the isolates [34]. However, the vaccine coverage rate of the isolates included in our study was slightly lower, estimated at 91.0%, highlighting the significant challenge of developing a vaccine that effectively addresses the extensive genetic diversity of GAS.

All GAS isolates analyzed in the present study were susceptible to penicillin and 75.1% of them were susceptible to all the tested antibiotics. From the introduction of penicillin as the first line treatment of GAS infections in clinical practice to this day, GAS has remained universally susceptible to β-lactam antibiotics [8, 45, 46]. However, in recent years, a few studies in GAS isolates have reported reduced susceptibility to β-lactams, primarily attributed to mutations in the gene that encodes penicillin binding protein 2X (*pbp2x*) [45].

In contrast to penicillin, 11.6% of isolates demonstrated resistance to macrolides, which is less than the previous seven-year data (2007–2013) from Greece (20.4%) with a significant annual variation [12]. In a more recent Greek study (2011–2017), a tendency for decrease in the macrolide resistance rate was observed, with the lowest rates occurring in years 2016 (5.5%) and 2017 (8.0%) [34]. This decrease could be attributed possibly to the reduction of macrolide consumption and/or the decreased circulation of macrolide-resistant GAS strains during the study period [34]. Moreover, the resistance rates have been also found to vary globally, with the rates estimated at 4–39% in Europe and in the US, and at > 40% in Asian countries [47]. A study conducted in Spain including iGAS and non-iGAS isolates collected between September 2022 and March 2023, found that macrolide resistance rate was 4.6% [48]. These findings suggest that macrolide resistance rate is temporally and geographically variable and is shaped by various factors such as clonal dynamics and antibiotic consumption [47].

In the present study, among the erythromycin-resistant isolates, 55.9% exhibited the M phenotype, 37.3% displayed the iMLS_B_ phenotype, and 6.8% presented the cMLS_B_ phenotype. In a previous study conducted in our region, the proportion of M-phenotype erythromycin-resistant isolates (53.7%) was comparable to our findings, whereas the prevalence of the iMLS_B_ (40.5%) and cMLS_B_ (5.8%) phenotypes showed slight differences [13].

Clindamycin is another antimicrobial agent appropriate for treating GAS infections in β-lactam allergic patients [49]. Additionally, clindamycin can be used as an adjunct therapy to β-lactam antibiotics in iGAS infections, as it has an antitoxin effect [46, 49]. However, concerns have been raised over the past few years, regarding the increasing rates of clindamycin resistance among GAS isolates [46, 50]. Reportedly, in the US, the rate of GAS clindamycin resistance raised from 0.5% in 2003 to as high as 15% in 2015 in pediatric populations [46, 51, 52]. In Greece, in two pediatric studies conducted in 2003–2006 and in 2007–2013, respectively, the clindamycin resistance rate among GAS isolates collected from children rose from 1.4% [13] to 13.8% [12]. In the current study, the rate of clindamycin resistance was lower than the earlier study (13.8%) [12] but still remained substantial, at 5.1%, emphasizing the need for caution when prescribing clindamycin until antimicrobial susceptibility is fully established [46]. In line with our findings, a recent study from Spain, conducted in 2022–2023 in the context of the GAS outbreak, reported a clindamycin resistance rate of 3.8%, in iGAS and non-iGAS isolates [48].

Limitations of this study include that only GAS strains isolated during 2023 were analyzed thus comparisons with previously circulated strains cannot be made. Additionally, non-iGAS isolates included from only two major cities and may not represent countywide epidemiology. NGS was performed only on fatal *emm1* isolates due to cost restrictions, and detailed clinical data were not available for all iGAS cases. However, this study included all the fatal and the majority of iGAS cases circulated in the whole country in the specific period and a robust sample of non-iGAS isolates. Importantly, the study represents the first in Greece to identify and report fatal M1_UK_ GAS infections in children.

A predominance of *emm12* and *emm1* were detected in non-iGAS isolates and of emm1 in iGAS, specifically M1_UK_ in fatal isolates. A decline in GAS macrolide resistance compared with previous studies in our area was detected. The findings of the present study suggest that future GAS vaccines must adapt to evolving epidemiological trends to ensure comprehensive protection against the most prevalent *emm* types in circulation. Epidemiological surveillance with molecular typing of circulating strains and monitoring resistance trends are required to ensure informing vaccine development and future preventive strategies aimed at reducing the burden of GAS-related diseases in children.

## Supplementary Information

Below is the link to the electronic supplementary material.

Supplementary Material 1

## Data Availability

No datasets were generated or analysed during the current study.
