# Supplementary material for: Molecular epidemiology and antimicrobial susceptibility of group A Streptococcus isolated from children in Greece during 2023
Source: Infection. 2025 Nov 3;54(1):155–68. doi: 10.1007/s15010-025-02639-0 (PMC12864330; doi:10.1007/s15010-025-02639-0)
Supplement: Supplementary file 1 — Supplementary Material 1 [file 15010_2025_2639_MOESM1_ESM.docx]

**Supplementary Material**

**Supplementary Table 1.** Age group distribution of *emm* types of Group A Streptococcus (GAS) isolates collected from children with invasive and non-invasive GAS infection from January to December 2023 in Greece.

| ***emm* type** | **Infants**  **(N=19)** | **Toddlers(N=68)** | **Pre-Schoolers**  **(N=179)** | **School-aged**  **Children**  **(N=198)** | **Adolescents**  **(N=32)** | **Total**  **(N=496)** |
| --- | --- | --- | --- | --- | --- | --- |
| ***emm1*** | 3 (15.8) | 23 (33.8) | 46 (25.7) | 52 (26.3) | 9 (28.1) | 133 (26.8) |
| ***emm2*** | 0 (0.0) | 0 (0.0) | 1 (0.6) | 5 (2.5) | 0 (0.0) | 6 (1.2) |
| ***emm3*** | 0 (0.0) | 0 (0.0) | 0 (0.0) | 4 (2.0) | 0 (0.0) | 4 (0.8) |
| ***emm4*** | 1 (5.3) | 0 (0.0) | 2 (1.1) | 2 (1.0) | 1 (3.1) | 6 (1.2) |
| ***emm8*** | 0 (0.0) | 0 (0.0) | 2 (1.1) | 0 (0.0) | 0 (0.0) | 2 (0.4) |
| ***emm11*** | 0 (0.0) | 0 (0.0) | 0 (0.0) | 1 (0.5) | 0 (0.0) | 1 (0.2) |
| ***emm12*** | 4 (21.1) | 30 (44.1) | 75 (41.9) | 81 (40.9) | 11 (34.4) | 201 (40.5) |
| ***emm22*** | 0 (0.0) | 0 (0.0) | 1 (0.6) | 1 (0.5) | 0 (0.0) | 2 (0.4) |
| ***emm28*** | 1 (5.3) | 1 (1.5) | 9 (5.0) | 9 (4.5) | 0 (0.0) | 20 (4.0) |
| ***emm33*** | 1 (5.3) | 0 (0.0) | 0 (0.0) | 1 (0.5) | 0 (0.0) | 2 (0.4) |
| ***emm43*** | 0 (0.0) | 0 (0.0) | 4 (2.2) | 1 (0.5) | 0 (0.0) | 5 (1.0) |
| ***emm44*** | 0 (0.0) | 0 (0.0) | 1 (0.6) | 0 (0.0) | 0 (0.0) | 1 (0.2) |
| ***emm49*** | 0 (0.0) | 0 (0.0) | 2 (1.1) | 0 (0.0) | 1 (3.1) | 3 (0.6) |
| ***emm60*** | 1 (5.3) | 0 (0.0) | 0 (0.0) | 1 (0.5) | 1 (3.1) | 3 (0.6) |
| ***emm63*** | 0 (0.0) | 1 (1.5) | 0 (0.0) | 0 (0.0) | 0 (0.0) | 1 (0.2) |
| ***emm68*** | 0 (0.0) | 0 (0.0) | 0 (0.0) | 1 (0.5) | 0 (0.0) | 1 (0.2) |
| ***emm75*** | 1 (5.3) | 5 (7.4) | 6 (3.4) | 8 (4.0) | 2 (6.3) | 22 (4.4) |
| ***emm76*** | 1 (5.3) | 0 (0.0) | 1 (0.6) | 2 (1.0) | 0 (0.0) | 4 (0.8) |
| ***emm77*** | 0 (0.0) | 0 (0.0) | 1 (0.6) | 3 (1.5) | 0 (0.0) | 4 (0.8) |
| ***emm81*** | 0 (0.0) | 0 (0.0) | 0 (0.0) | 0 (0.0) | 1 (3.1) | 1 (0.2) |
| ***emm82*** | 1 (5.3) | 0 (0.0) | 0 (0.0) | 0 (0.0) | 0 (0.0) | 1 (0.2) |
| ***emm83*** | 0 (0.0) | 0 (0.0) | 0 (0.0) | 1 (0.5) | 0 (0.0) | 1 (0.2) |
| ***emm87*** | 0 (0.0) | 0 (0.0) | 2 (1.1) | 2 (1.0) | 0 (0.0) | 4 (0.8) |
| ***emm89*** | 3 (15.8) | 5 (7.4) | 14 (7.8) | 14 (7.1) | 2 (6.3) | 38 (7.7) |
| ***emm104*** | 1 (5.3) | 0 (0.0) | 0 (0.0) | 3 (1.5) | 0 (0.0) | 4 (0.8) |
| ***emm118*** | 0 (0.0) | 0 (0.0) | 0 (0.0) | 1 (0.5) | 1 (3.1) | 2 (0.4) |
| ***emm128*** | 1 (5.3) | 3 (4.4) | 10 (5.6) | 5 (2.5) | 1 (3.1) | 20 (4.0) |
| ***emm168*** | 0 (0.0) | 0 (0.0) | 0 (0.0) | 0 (0.0) | 1 (3.1) | 1 (0.2) |
| ***emm223*** | 0 (0.0) | 0 (0.0) | 0 (0.0) | 0 (0.0) | 1 (3.1) | 1 (0.2) |
| ***emm227*** | 0 (0.0) | 0 (0.0) | 1 (0.6) | 0 (0.0) | 0 (0.0) | 1 (0.2) |
| ***emm228*** | 0 (0.0) | 0 (0.0) | 1 (0.6) | 0 (0.0) | 0 (0.0) | 1 (0.2) |

**Notes:** Values are referred as absolute frequencies (relative frequencies. %

**Supplementary Table 2.** Seasonal distribution of the *emm* types of Group A Streptococcus (GAS) isolates collected from children with invasive and non-invasive GAS infection from January to December 2023 in Greece.

| ***emm* type** | **Winter**  **(N=81)** | **Spring**  **(N=182)** | **Summer**  **(N=170)** | **Autumn**  **(N=77)** | **Total (N=510)** |
| --- | --- | --- | --- | --- | --- |
| ***emm1*** | 34 (42.0) | 36 (19.8) | 45 (26.5) | 22 (28.6) | 137(26.9) |
| ***emm2*** | 4 (4.9) | 0 (0.0) | 2 (1.2) | 0 (0.0) | 6 (1.2) |
| ***emm3*** | 2 (2.5) | 1 (0.5) | 1 (0.6) | 0 (0.0) | 4 (0.8) |
| ***emm4*** | 2 (2.5) | 0 (0.0) | 2 (1.2) | 2 (2.6) | 6 (1.2) |
| ***emm8*** | 0 (0.0) | 1 (0.5) | 0 (0.0) | 1 (1.3) | 2 (0.4) |
| ***emm11*** | 0 (0.0) | 1 (0.5) | 0 (0.0) | 0 (0.0) | 1 (0.2) |
| ***emm12*** | 10 (12.3) | 94 (51.6) | 84 (49.4) | 21 (27.3) | 209 (41.0) |
| ***emm22*** | 1 (1.2) | 0 (0.0) | 1 (0.6) | 0 (0.0) | 2 (0.4) |
| ***emm28*** | 7 (8.6) | 9 (4.9) | 2 (1.2) | 2 (2.6) | 20 (3.9) |
| ***emm33*** | 0 (0.0) | 0 (0.0) | 0 (0.0) | 2 (2.6) | 2 (0.4) |
| ***emm43*** | 0 (0.0) | 2 (1.1) | 2 (1.2) | 1 (1.3) | 5 (1.0) |
| ***emm44*** | 0 (0.0) | 0 (0.0) | 0 (0.0) | 1 (1.3) | 1 (0.2) |
| ***emm49*** | 1 (1.2) | 0 (0.0) | 2 (1.2) | 0 (0.0) | 3 (0.6) |
| ***emm60*** | 0 (0.0) | 1 (0.5) | 0 (0.0) | 2 (2.6) | 3 (0.6) |
| ***emm63*** | 0 (0.0) | 0 (0.0) | 0 (0.0) | 1 (1.3) | 1 (0.2) |
| ***emm68*** | 1 (1.2) | 0 (0.0) | 0 (0.0) | 0 (0.0) | 1 (0.2) |
| ***emm73*** | 0 (0.0) | 0 (0.0) | 0 (0.0) | 1 (1.3) | 1 (0.2) |
| ***emm75*** | 7 (8.6) | 4 (2.2) | 8 (4.7) | 4 (5.2) | 23 (4.5) |
| ***emm76*** | 1 (1.2) | 1 (0.5) | 0 (0.0) | 2 (2.6) | 4 (0.8) |
| ***emm77*** | 0 (0.0) | 1 (0.5) | 1 (0.6) | 2 (2.6) | 4 (0.8) |
| ***emm81*** | 0 (0.0) | 1 (0.5) | 0 (0.0) | 0 (0.0) | 1 (0.2) |
| ***emm82*** | 0 (0.0) | 0 (0.0) | 0 (0.0) | 1 (1.3) | 1 (0.2) |
| ***emm83*** | 0 (0.0) | 0 (0.0) | 1 (0.6) | 0 (0.0) | 1 (0.2) |
| ***emm87*** | 0 (0.0) | 2 (1.1) | 1 (0.6) | 1 (1.3) | 4 (0.8) |
| ***emm89*** | 8 (9.9) | 12 (6.6) | 11 (6.5) | 7 (9.1) | 38 (7.5) |
| ***emm104*** | 0 (0.0) | 0 (0.0) | 1 (0.6) | 3 (3.9) | 4 (0.8) |
| ***emm118*** | 0 (0.0) | 1 (0.5) | 1 (0.6) | 0 (0.0) | 2 (0.4) |
| ***emm128*** | 2 (2.5) | 14 (7.7) | 3 (1.8) | 1 (1.3) | 20 (3.9) |
| ***emm168*** | 0 (0.0) | 0 (0.0) | 1 (0.6) | 0 (0.0) | 1 (0.2) |
| ***emm223*** | 1 (1.2) | 0 (0.0) | 0 (0.0) | 0 (0.0) | 1 (0.2) |
| ***emm227*** | 0 (0.0) | 0 (0.0) | 1 (0.6) | 0 (0.0) | 1 (0.2) |
| ***emm228*** | 0 (0.0) | 1 (0.5) | 0 (0.0) | 0 (0.0) | 1 (0.2) |

**Notes:** Values are referred as absolute frequencies (relative frequencies. %).

**Supplementary Table 3.** Genetic and antimicrobial resistance profile of the *emm1* Group A Streptococcus (GAS) isolates collected from fatal cases.

| **GAS isolate** | **SRA biosample code** | **Isolation Source** | ***emm* type** | ***emm* Subtype** | **Cluster** | **M1 lineage** | **Superantigen genes** | **AST** | **AMR**  **genes** | **Host’s**  **Age**  **(years)** | **Host’s**  **Gender** | **Type of Streptococcal infection** |
| --- | --- | --- | --- | --- | --- | --- | --- | --- | --- | --- | --- | --- |
|  |  |  |  |  |  |  |  | **Resistant**  **(MIC)** |  |  |  |  |
| 1 | SAMN48785444 | Blood | *emm1* | *emm1.0* | A-C3 | M1_UK_ | *smeZ-speA-speG-speJ* | TET (3), MOX (1.5), RIF (0.25) | Not detected | 6.8 | Female | Bacteremia, STSS |
| 2 | SAMN48801584 | Blood | *emm1* | *emm1.0* | A-C3 | M1_UK_ | *speA* | NO | Not detected | 1.7 | Male | Bacteremia, pneumonia |
| 3 | SAMN48787133 | Blood | *emm1* | *emm1.0* | A-C3 | M1_UK_ | *smeZ-speA-speG-speJ* | ERY (0.5) | Not detected | 2.7 | Male | Bacteremia, STSS |
| 4 | SAMN48800132 | Blood | *emm1* | *emm1.61* | A-C3 | M1_global_ | *smeZ-speA-speG-speJ* | NO | Not detected | 2.5 | Male | Bacteremia, STSS |
| 5 | SAMN48801396 | Blood | *emm1* | *emm1.0* | A-C3 | M1_UK_ | *smeZ-speA-speC-speG-speJ* | NO | Not detected | 1.1 | Female | Bacteremia, pneumonia, STSS |
| 6 | SAMN48787878 | Blood | *emm1* | *emm1.0* | A-C3 | M1_UK_ | *smeZ-speA-speG-speJ* | NO | Not detected | 1.8 | Male | Bacteremia, pneumonia |
| 7 | SAMN48801536 | Blood | *emm1* | *emm1.0* | A-C3 | M1_UK_ | *smeZ-speA-speG-speJ* | NO | Not detected | 8.1 | Male | Bacteremia, STSS |

**Abbreviations:** AST; Antimicrobial susceptibility testing, SRA: Sequence Read Archive (SRA), TET; Tetracycline, MOX; Moxifloxacin, RIF; Rifampicin, AMR; Antimicrobial resistance, STSS; Streptococcal toxic shock syndrome
